# Total centromere size and genome size are strongly correlated in ten grass species

**DOI:** 10.1007/s10577-012-9284-1

**Published:** 2012-05-03

**Authors:** Han Zhang, R. Kelly Dawe

**Affiliations:** 1 Department of Genetics, University of Georgia, Athens, GA 30602 USA; 2 Department of Plant Biology, University of Georgia, Miller Plant Sciences Bldg., Athens, GA 30602 USA

**Keywords:** centromere size, chromosome length, genome size, chromosome number, kinetochore size, grasses

## Abstract

It has been known for decades that centromere size varies across species, but the factors involved in setting centromere boundaries are unknown. As a means to address this question, we estimated centromere sizes in ten species of the grass family including rice, maize, and wheat, which diverged 60~80 million years ago and vary by 40-fold in genome size. Measurements were made using a broadly reactive antibody to rice centromeric histone H3 (CENH3). In species-wide comparisons, we found a clear linear relationship between total centromere size and genome size. Species with large genomes and few chromosomes tend to have the largest centromeres (e.g., rye) while species with small genomes and many chromosomes have the smallest centromeres (e.g., rice). However, within a species, centromere size is surprisingly uniform. We present evidence from three oat–maize addition lines that support this claim, indicating that each of three maize centromeres propagated in oat are not measurably different from each other. In the context of previously published data, our results suggest that the apparent correlation between chromosome and centromere size is incidental to a larger trend that reflects genome size. Centromere size may be determined by a limiting component mechanism similar to that described for *Caenorhabditis elegans* centrosomes.

**Electronic supplementary material:**

The online version of this article (doi:10.1007/s10577-012-9284-1) contains supplementary material, which is available to authorized users.

## Introduction

Centromeres are the chromosomal domains responsible for accurate chromosome segregation during mitosis and meiosis. In higher eukaryotes, centromeres are characterized by long segments of tandem repeats. Although these arrays extend several megabases in plants and animals, only a fraction of the repetitive sequences are incorporated into centromeric chromatin, which is specified by the specialized histone H3 variant CENH3 (Blower et al. 2002; Zhong et al. 2002). CENH3 and its distinct chromatin environment are directly responsible for recruiting the overlying kinetochore proteins that ultimately interact with microtubules (Cheeseman and Desai 2008). The number of attached microtubules is species specific and ranges from one to eighty microtubules/kinetochore (Peterson and Ris 1976; Jensen 1982). In the budding yeast *Saccharomyces cerevisiae*, which has the smallest known centromere at 125 bp, a single CENH3 nucleosome is linked to a single spindle microtubule through a protein linkage consisting of one to two copies of CENPC, six to seven copies of the MIS12 complex, and eight copies of the NDC80 complex (Joglekar et al. 2006). Fission yeast centromeres have two to three CENH3 nucleosomes and interact with two to four microtubules (Joglekar et al. 2008). However, it is unlikely that this simple stoichiometric relationship can be applied to large genome species such as human, where there appears to be thousands of CENH3 nucleosomes in each centromere (Black et al. 2007) but only ~17 microtubules per kinetochore (McEwen et al. 2001).

It is natural to wonder if large centromeres are a consequence of the logistics of pulling large chromosomes. In animal lineages, on a species-wide scale, centromere size does appear to correlate with chromosome size. Using broadly reactive anti-kinetochore (CREST) antisera, previous authors showed that species with few large chromosomes have larger centromeres than species with numerous small chromosomes (Cherry et al. 1989). In contrast, comparisons of individual centromeres within a species reveal only minor differences in size that are independent of chromosome length, a distinct lack of a trend (Cherry and Johnston 1987; Fantes et al. 1989; Schmitz et al. 1992). A more recent study used a fluorescent CENH3 fusion protein in human cultured cells to reveal a weak (40 %) correlation between chromosome size and quantity of CENH3 (Irvine et al. 2004). Taken together, the available animal data suggest that both chromosome number and chromosome size may impact the size of centromeres. There have been no similar studies in plants, or particularly the grasses, where there is less variation in basic chromosome number but dramatic differences in chromosome size. For instance, haploid rice and rye has 12 and 7 chromosomes respectively but vary more than 30-fold in DNA content (Matsumoto et al. 2005; Bartos et al. 2008). There have also been several rounds of genome duplication in some lineages, including cultivated wheat, which has a hexaploid genome that originated through two polyploidization events (Salse et al. 2008).

The mechanisms controlling centromere size may involve changes to the underlying DNA sequences, or epigenetic events that do not involve changes in DNA. In human cells, the amount of CENH3 is proportional to the amount of centromeric satellite repeats, suggesting that expansions or contractions of satellite arrays may directly affect the size of the functional centromere cores (Sullivan et al. 2011). However, this is not true in rice, where the lengths of rice centromeric satellite arrays vary by nearly 30-fold among chromosomes but the CENH3 staining area is not nearly this variable (Yan et al. 2008). Epigenetic control of centromere size has been demonstrated in the yeast *Candida albicans*, where CENH3 overexpression causes CENH3 nucleosomes to replace adjacent H3-containing nucleosomes, increasing the number of kinetochore proteins and spindle microtubules (Burrack et al. 2011). Centromere size plasticity has also been reported in an oat strain containing a fragment of a maize chromosome with a newly created centromere. The size of the CENH3 domain on the maize neocentromere ranged from 30 to 90 % of the oat centromeres depending on the tissue assayed (Topp et al. 2009). The authors suggest that broken or newly established centromeres may expand over flanking regions, demonstrating their inherent capacity to adjust to new genetic backgrounds.

Here, we report the results of immunofluorescence experiments using an anti-OsCENH3 antibody that functions in multiple grass species (Jin et al. 2004; Nagaki et al. 2004; Liu et al. 2008; Sanei et al. 2011). By comparing species that vary substantially in karyotype, we show that the size of the CENH3 domains correlate strongly with genome size but not necessarily chromosome size.

## Materials and methods

### Materials

Seeds of oat (PI 502922), barley (PI 539128), rye (PI 534936), sorghum (PI 564163), *Zea luxurians* (PI422162), and maize B73 inbred (PI550473) were ordered from USDA-ARS. Seeds of wheat (Chinese Spring), foxtail millet (Yugul), pearl millet (Tift23db), and rice were generously provided by Katrien M. Devos. Oat–maize addition lines OMA 2.01, 6.01, and 9.01 were a gift from Dr. Howard Rines, University of Minnesota.

### Cytological preparation and observation

Protein sequences of the amino-terminal of CENH3 were acquired from the Genebank: maize (accession number NP_001105520), rice (accession number AAR85315), barley (accession number AEK21392), and oat (accession number ACI01453). Sequences were aligned using the ClustalW2 software (http://www.ebi.ac.uk/Tools/msa/clustalw2/).

Seeds from different species were germinated on wet paper towels for 3 days to a week depending on the species. Root tips were fixed in 4 % paraformaldehyde diluted in PBS buffer for 30 min and stored in methanol if not directly used for immunolocalization. After fixation, root tips were digested in an enzyme mix containing 4 % cellulase, 2 % pectolyase in 10 mM Citric Buffer, pH 5.5, for 20 min to an hour at 37 °C. Digested root tip cells were rinsed in PBS buffer, and then dropped on the slides and centrifuged at 100×*g* for 1 min. Slides were incubated with anti-OsCENH3 antibody (1:200, Nagaki et al. 2004), anti-maize CENH3 antibody (1:100, Zhong et al. 2002), anti-oat CENH3 antibody (1:100, Topp et al. 2009), or anti-tubulin antibody (1:500, Asai et al. 1982) overnight at 4 °C, followed by an hour blocking with goat serum (1:10) and 2-h incubation with secondary antibodies (1:200) at room temperature. The slides were then stained with 0.1 mg/ml 4,6-diamidino-2-phenylindole (DAPI). All images were captured and processed using a Zeiss Axio Imager microscope (http://www.zeiss.com/) and SlideBook 5.0 software (Imaging Innovations, https://www.intelligent-imaging.com/).

To identify the maize chromosomes in the oat background, cells were first subject to the same protocol as for immunolocalization. The mounted cells were rinsed for ten minutes in PBS buffer, post-fixed with 4 % paraformaldehyde diluted in PBS buffer (to fix the antibodies at the site of binding), and then rinsed again in PBS before performing FISH. FISH was performed using maize centromere-specific CRM probes (Shi et al. 2010) according to the protocols described previously (Kato et al. 2004), except that the salmon sperm DNA and the probes were mixed together and applied to the slides before denaturing in situ.

### Centromere measurement and statistical analysis

Fluorescent signals were captured as 3D images using the Digital Microscope Workstation as previously described (Du and Dawe 2007). A projection image was made for each capture and deconvolved using SlideBook software to reduce background interference. Centromere signals were measured using the mask tool to select regions of interest. A threshold was set at one intensity unit above the brightest non-centromeric staining in the cell. The total area covering the selected regions was measured in at least 20 cells for each species. Microsoft Excel software was used to calculate the average centromere area and standard deviation for each species. Genome size and chromosome number of the grass species were acquired from the Gramene database (http://www.gramene.org/species/). Linear regression and correlation coefficients were calculated using Excel software.

To determine whether the correlation between genome size and total centromere area was influenced by phylogeny, independent contrast was applied using the COMPARE 4.6b software (http://www.indiana.edu/~martinsl/compare/) with the phylogenetic tree shown in Supplemental Fig. 2.

When measuring CENH3 in oat–maize additional lines, the maize centromeres were identified by the overlapping CRM FISH signals. Oat centromere values were calculated as the average staining from the remaining centromeres. The resulting maize and oat centromere size data from all cells were compared by standard *t* tests.

## Results

### CENH3 domains vary in size across species

**Fig. 1:**
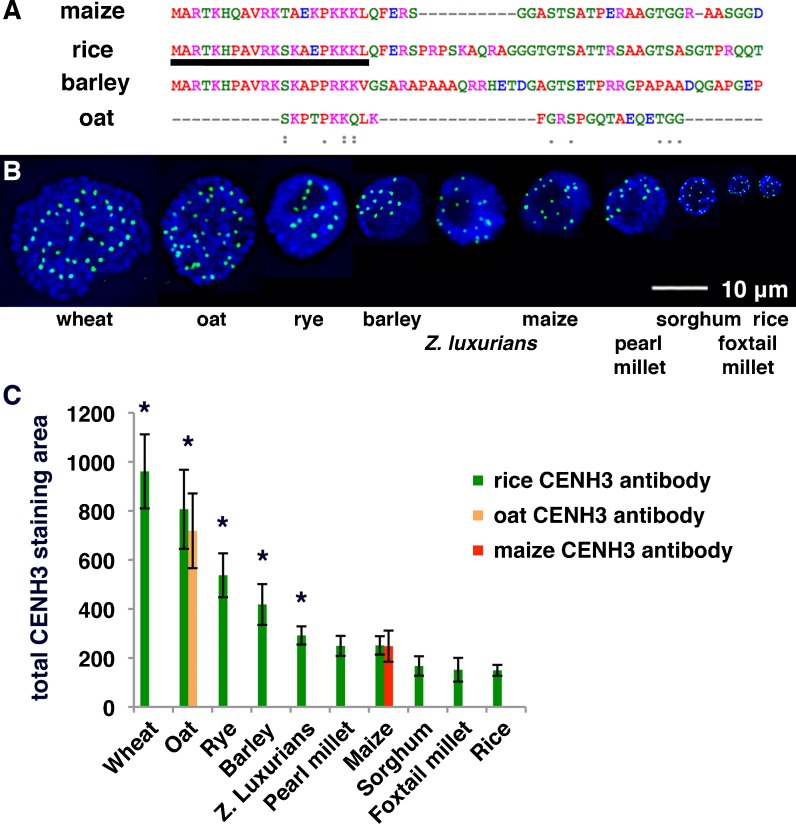
Centromere size variation in the grass species. **a** ClustalW2 alignment of the extreme amino-terminal sequences of CENH3 proteins in the grass species. The sequence from which the anti-OsCENH3 antibody was generated is *underlined*. **b** Immunofluorescence images showing that the kinetochores differ in size among species. Interphase cells of varied grass species were stained with anti-OsCENH3 antibodies (*green*) and DAPI (*blue*). **c** Total centromere areas for ten species in the grass family. Species with centromere areas that are significantly different (*p* < 0.01) from all others are indicated with *stars*. CENH3 intensity units are arbitrary and only relevant to each other

In order to examine centromere size variation, we assayed CENH3 in ten species of the grass family using an antibody that recognizes the extreme amino terminus of the rice CENH3 protein (Nagaki et al. 2004). This section of the CENH3 protein is relatively well conserved in the cereal grasses (Fig. 1a), and the antibody is known to recognize CENH3 in rice, maize, oat, sugarcane, barley, wheat, and rye (Jin et al. 2004; Nagaki et al. 2004; Nagaki and Murata 2005; Houben et al. 2007; Liu et al. 2008; Schubert et al. 2011). We found that the antibody also identifies centromeres in *Zea luxurians*, pearl millet, foxtail millet, and sorghum (Fig. 1b). The broad reactivity of the antibody makes it a valuable tool for comparing centromere size across species, as has been done previously in animal cells using human CREST antisera (Cherry et al. 1989).

To minimize the affects of variable antibody affinity in heterologous species, centromere size was estimated from staining area (not intensity) in two-dimensional projection images (see Materials and Methods). For each image, a threshold was set at one intensity unit above the brightest non-centromere staining in the cell (Supplemental Fig. 1). Although exposure time does not influence the values obtained by this method, antibody affinity may, since low antibody affinity can lead to high background staining. To test the general reliability of this method, we measured centromeres using an antibody that specifically recognizes maize CENH3 and another that specifically recognizes oat CENH3 (Zhong et al. 2002; Topp et al. 2009). Measurements obtained using species-specific antisera were nearly identical to the values obtained using the heterologous rice antibody (Fig. 1c), suggesting that our measurements provide reliable estimates of total centromere size. By this measure, each species has a total centromere area that is statistically different from others, except that pearl millet is indistinguishable from maize and sorghum is indistinguishable from foxtail millet and rice (Fig. 1c).

### Total CENH3 staining is correlated with genome size

**Fig. 2:**
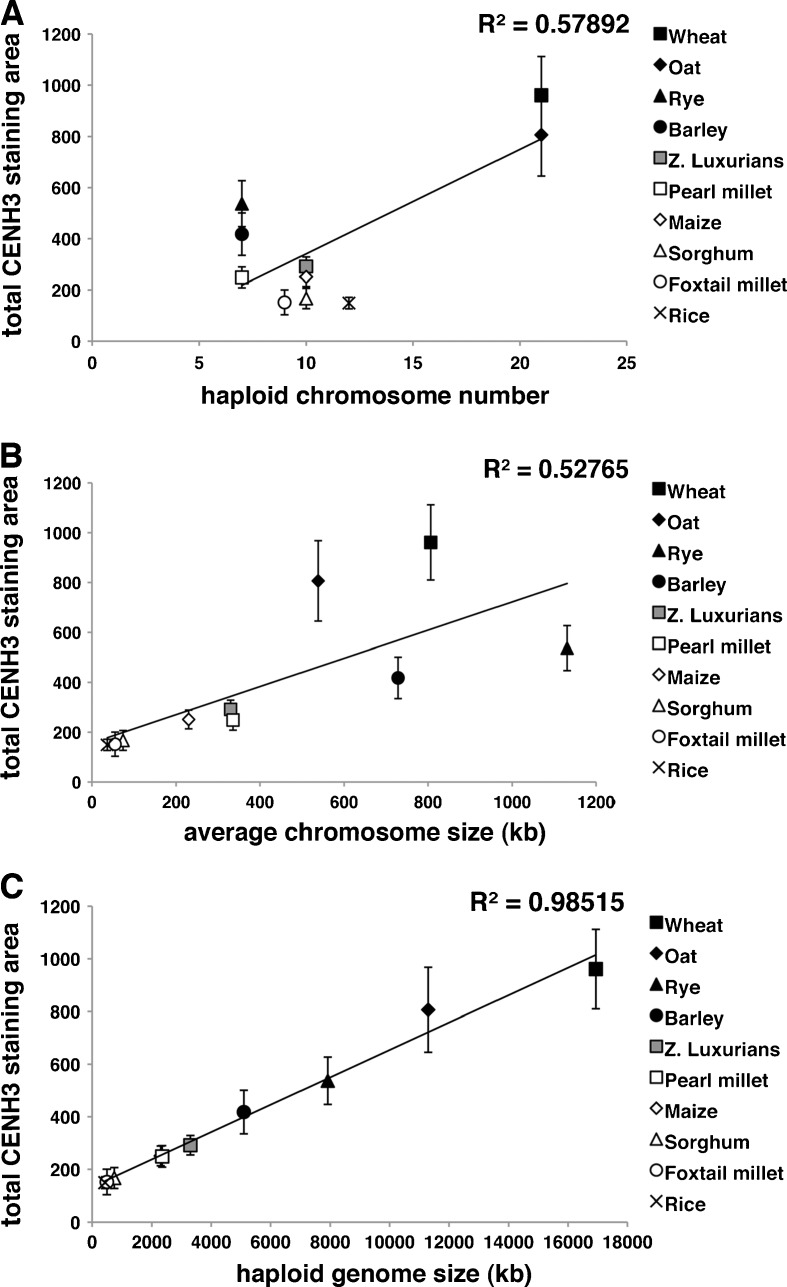
Relationship between total centromere area and chromosome number, chromosome size, and genome size. **a** Correlation between total centromere area and chromosome number. **b** Correlation between total centromere area and average chromosome size. **c** Correlation between total centromere area and genome size

Prior data from mammalian cells suggest that some lineages show a correlation between kinetochore size and chromosome number (Cherry et al. 1989). Our data are well suited to a similar analysis since the ten species chosen vary from 7 to 21 chromosomes per haploid complement (http://www.gramene.org/species/). Plotting our CENH3 measurements against chromosome number revealed a moderate but significant correlation (Fig. 2a, *R* ^2^ = 0.579, *p* < 0.05). We also considered that chromosome size could be a better indicator of centromere size, since larger chromosomes might reasonably be expected to require larger centromeres to guide them through cell division. The great majority of chromosome size variation in the grasses occurs among species (>30-fold) while within species, chromosome size varies by smaller magnitudes (~2- to 3-fold) (Matsumoto et al. 2005; Paterson et al. 2009; Schnable et al. 2009). We plotted total CENH3 staining against average chromosome size for each species (as calculated by genome size divided by chromosome number). This comparison again revealed a moderate but significant correlation, suggesting that chromosome size can explain about 52.8 % of the variation in CENH3 staining (*R* ^2^ = 0.528, *p* < 0.05; Fig. 2b).

We next investigated the assumption that total CENH3 staining area may be correlated with total genome size. This analysis revealed a 98.5 % correlation between the two variables (*p* < 0.01; Fig. 2c). Correlations such as this can be strongly affected by evolutionary context; for instance, in cases where species cluster into closely related subgroups the correlations may be artificially elevated (Felsenstein 1985). To test for such artifacts, the same regression analysis was performed under conditions weighted by evolutionary context (Martins 2004) using a phylogeny based on the trnL-trnF intergenic spacer of the chloroplast genome (Drabkova et al. 2004). This modification tightened the correlation, indicating that 99 % of total variation in CENH3 staining can be explained as a function of genome size (data not shown).

**Fig. 3:**
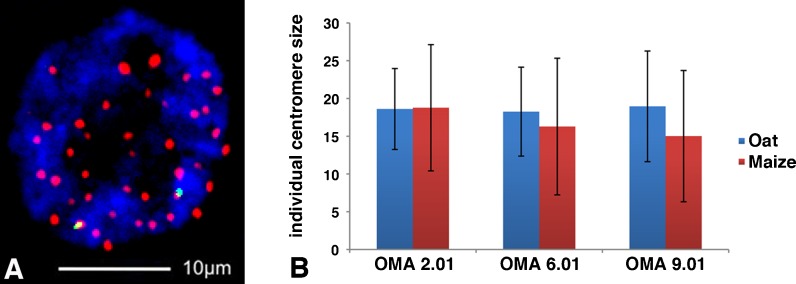
Comparison of centromere staining areas in the oat–maize addition lines. **a** An immunoFISH image of an oat–maize addition line showing that maize centromeres (as identified by the *green* maize-specific CRM probes) are not significantly smaller than oat centromeres. The cell was stained with anti-OsCENH3 antibodies (*red*), CRM probes (*green*), and DAPI (*blue*). **b** The staining areas of maize centromeres 2 (*n* = 28), 6 (*n* = 12), and 9 (*n* = 27) are compared with each other and the average of the oat centromeres in the corresponding cells

Our measurements suggest that centromere size is regulated at the whole cell level and reflects genome size rather than chromosome size. To test this hypothesis, we studied oat–maize addition lines retaining maize chromosomes of different lengths (Ananiev et al. 1997; Kynast et al. 2004). Oat lines containing maize chromosomes 2, 6, and 9 were assayed independently using a protocol that combines immunofluorescence (for CENH3) and FISH (for the maize chromosomes). If the size of the maize centromere reflects the size of the chromosome where it is located, we would expect that the introduced maize centromeres would differ from each other and be significantly different from the naturally larger oat centromeres (Fig. 1c). If centromere size is controlled primarily at the cellular level, we would expect the maize and oat centromeres to have similar sizes in the same cell. The combined data from three different lines generally support the view that centromere size is independent of chromosome size, given that the maize centromeres are indistinguishable from each other and from average oat centromeres in the same cells (Fig. 3). However, we found that the immuno-FISH procedure lacks sufficient reproducibility on a single-centromere scale (Fig. 3a and error bars in b) to definitively accept or reject this hypothesis.

### Relationship between kinetochore size and microtubule number

**Fig. 4:**
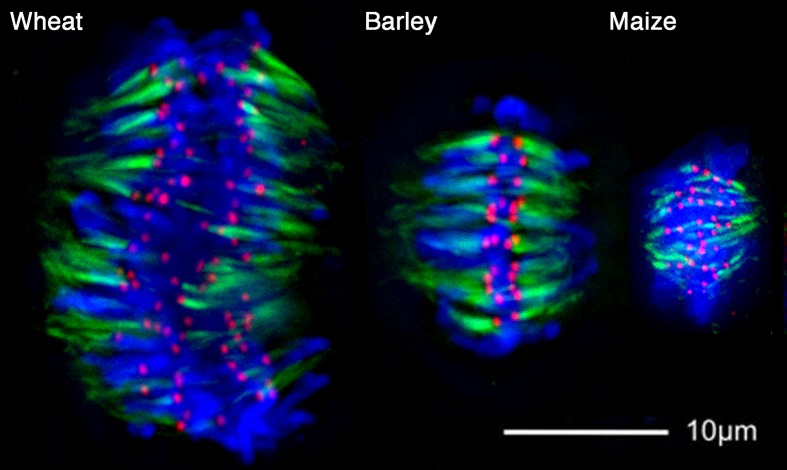
Comparison of microtubule and kinetochore staining in three grass species. Metaphase chromosomes were stained with anti-OsCENH3 antibodies (*red*), an anti-tubulin antibody (*green*), and DAPI (*blue*). Note that microtubules are more abundant in species with large centromeres (wheat and barley) and less abundant in species with small centromeres (maize)

To confirm that there is a positive correlation between size of the CENH3 domains and the number of microtubule attachments on grass kinetochores, we performed immunofluorescence using a widely-reactive anti-tubulin antibody (Asai et al. 1982). Consistent with data from other species, our results reveal that the density of microtubules is much higher in species with larger CENH3 domains (e.g., wheat and barley) than those with small CENH3 domains (e.g., maize; Fig. 4).

## Discussion

All eukaryotes have centromeres of a characteristic size and content, determined by the centromere identifier CENH3. Measuring centromere size by direct sequencing is only possible in species with few or frequently interrupted satellite repeats (Yan et al. 2008; Wolfgruber et al. 2009). In contrast, measuring centromere size by immunofluorescence is less technically demanding and has been performed in several previous studies (Cherry and Johnston 1987; Cherry et al. 1989; Fantes et al. 1989; Schmitz et al. 1992). The success of this method relies on the availability of an antibody that recognizes centromeres across multiple related species. Such antibodies are particularly rare for CENH3 because the protein is variable by nature (Henikoff et al. 2001). Grasses are unusual in having a conserved motif at the far N terminus of CENH3 (Talbert et al. 2004), and an antibody raised against this region has proven to have broad reactivity (Nagaki et al. 2004). The ten species selected for this study diverged 60~80 million years ago and represent much of the diversity in the true grasses (Bennetzen and Freeling 1997; Soreng and Davis 1998).

Our measurements using anti-CENH3 antibodies reveal that each grass species has a characteristic centromere size (Fig. 1c) that is strongly correlated with genome size (Fig. 2c). In rice, centromeres have been estimated to span physical distances ranging 420–820 kb, although there are often long intervening regions containing canonical H3 within the centromere cores (Yan et al. 2008). Two sequenced and fully assembled maize centromeres (from chromosomes 2 and 5) contain CENH3-rich areas spanning 1.8 and 4.2 Mb, respectively (Wolfgruber et al. 2009). Given that centromere size closely follows genome size, it is likely that many plant species contain centromeres that exceed 10 Mb. Our data also suggest a minimum centromere size since the graphs do not appear to intersect in zero (Fig. 2c); that is, a genome of zero kilobases appears to have a non-zero centromere size. At least one CENH3 nucleosome is required to move any chromosome, but the minimum centromere size is probably much larger in plants.

It is tempting to extend the trend between genome size and centromere size to the chromosome level and suggest that the larger chromosomes within a species might also have larger centromeres. Rice chromosomes vary about 2-fold in size from the smallest to the largest, but centromere size does not appear to follow this trend (Yan et al. 2008). Animal chromosomes vary more dramatically in size though there is little evidence that larger chromosomes have larger centromeres. In chicken DT40 cells, so-called macrochromosomes are ~20 times larger than the minichromosomes, but their centromeres appear to be roughly the same size (Johnston et al. 2010). Another meaningful estimate of centromere size is the number of microtubules attached at metaphase. Prior authors have painstakingly measured kinetochore microtubules in a series of organisms (Ding et al. 1993; Winey et al. 1995; McEwen et al. 1998, 2001; Joglekar et al. 2008; Gan et al. 2011). A tabulation of these published data (Table 1) reveals a clear positive relationship between microtubule number and average chromosome size among species (*R* ^2^ = 0.92, *p* < 0.01) that parallels our observations using CENH3 staining. However, within species, these authors did not observe any correlation between the size of the chromosome and the number of attached microtubules. For instance, in three species of grasshoppers, chromosome volume varies up to 10-fold with little or no increase in microtubule numbers (Moens 1979). We made similar observations in the plant species assayed here (Fig. 4) showing that though larger centromeres tend to associate with more microtubules, there is no obvious variation in the size of the microtubule bundles between chromosomes.

**Table 1:** Kinetochore microtubule numbers are related to average chromosome size in multiple species

	Species	Haploid genome size^a^ (Mb)	Haploid chromosome number	Average chromosome size (Mb)	Microtubule number per kinetochore	References
Yeasts	*Schizosaccharomyces pombe*	14.1	3	4.7	3	Ding et al. 1993
	*Saccharomyces cerevisiae*	12	16	0.8	1	Winey et al. 1995
	*Candida albicans*	16	8	2.0	1	Joglekar et al. 2008
Animals	*Drosophila*	165	4	41.3	5	McEwen et al. 1998
	Human	3,000	23	130.4	17	McEwen et al. 2001
	Fetal rats	2,800	21	133.3	7	McEwen et al. 1998
	CHO cells	3,032	11	275.6	12	McEwen et al. 1998
	PtK cells	3,000	6	500.0	24	McEwen et al. 1998
Alga	*Ostreococcus tauri*	12	20	0.6	0.4	Gan et al. 2011
Plant	*Haemanthus*	57,213	8	7,151.6	75	McEwen et al. 1998

^a^Haploid genome size and chromosome number were obtained from the following databases: fungal, www.zbi.ee/fungal-genomesize/; animals, www.genomesize.com; and plant and algae, www.kew.org/genomesize/homepage.html

**Fig. 5:**
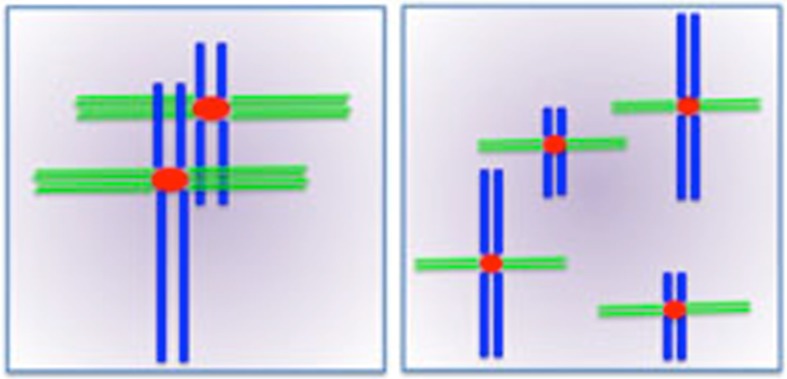
A limiting component model for the control of centromere size. The model shows that each species has a total centromere area that relates to genome size. A genome with few chromosomes will generally have large centromeres (*left*); however, if the same genome contained more chromosomes, the centromeres would be smaller (*right*). The total centromere area would be same in either case

To explain the observation that total centromere size varies among species according to genome size but is independent of within-species chromosome size, we propose the model shown in Fig. 5. A key assumption of our model is that DNA content positively correlates with cell and nuclear volume (Price et al. 1973; Szarski 1976; Jovtchev et al. 2006). We propose that for each species there is a total centromere area required to stabilize the spindle. A genome with few chromosomes will generally have large individual centromeres, but the total centromere area (i.e., total number of CENH3 nucleosomes) would be unchanged if the same genome were divided into many chromosomes with small centromeres (Fig. 5). Our model is very similar to a recently proposed limiting component model for the control of centrosome size in *Caenorhabditis elegans* (Decker et al. 2011). Under conditions where a key centrosome precursor was limiting, centrosomal area was dependent on both cell volume and the number of centrosomes in the cell: large cells have large centrosomes unless the centrosome is divided in two, in which case each centrosome was half the size of the original. This model may help to explain the sizes of many organelles, including fundamental structures such as the nucleus and cytoplasm (Decker et al. 2011; Marshall 2011). Under the limiting component model, centromere size varies naturally with chromosome size not because large chromosomes require more force to move but because the cells that house large chromosomes must build large spindles to ensure the complete separation of the chromosomes at anaphase.

We have begun to test this hypothesis by studying the results of a cross between two species with different centromere sizes. Such a cross was made previously between maize and oat (Rines et al. 1995). Maize centromeres are half as large as oat centromeres in their native state (Fig. 1c), but when three different maize chromosomes were added to oat and maintained for several generations, this size difference was not apparent (Fig. 3b). We view these data as preliminary due to the high sampling error when measuring single centromeres in our assay. However, it will be possible to further test this hypothesis on maize centromeres 2 and 5, which are sequenced and can be compared in the maize and oat backgrounds using CENH3 chromatin immunoprecipitation. The inherent flexibility in the lengths of CENH3 domains suggests that centromeres are likely to change when placed in a different species. In the case of the oat–maize addition lines, the most likely outcome will be that the maize centromeres will adapt to the larger oat genome and increase in size (Topp et al. 2009).

## Electronic supplementary material

Below is the link to the electronic supplementary material.

Supplemental Fig. 1 Illustration of the masking protocol used to determine total centromere area. A single image from barley is shown after masking at various threshold values. CENH3 is shown in *green* and DAPI is shown in *blue*. **a** Original unmasked image. **b**–**e** Images shown after masking at progressively higher threshold values so that more and more of the staining is removed. The image in (**d**) shows the threshold chosen, which was set at one grey level above that needed to remove all non-kinetochore background staining (JPEG 8 kb)

High resolution image (TIFF 2,176 kb)

Supplemental Fig. 2 Phylogenetic tree showing the relationship of the grass species chosen in this study. The phylogenetic tree was drawn using the following Newick tree: (((sorghum, 0.00950939280685307413 (maize, 0.00231520485691936832; Z. luxurians, 0.01052332533144828206), 0.00367176807056998015), 0.01041711803339982756 (foxtail millet, 0.01363691334023187443; pearl millet, 0.01520671944747864317), 0.01592780163032410726), 0.02586075002500199532 ((oat, 0.03072769607026095903 (barley, 0.01983366653899813412 (rye, 0.01449881409177665101; wheat, 0.00350123323061236674), 0.00909150693055843159), 0.02146457938246661493), 0.01807421402216232917; rice, 0.04736460143268115403), 0.00833952027397439483). The same tree was applied to perform independent contrast using the COMPARE 4.6b software (GIF 8 kb)

High resolution image (EPS 17,501 kb)

## Abbreviations

CENH3: Centromere histone H3
CENPC: Centromere protein C
MIS12: Minichromosome instability 12
Ndc80: Nuclear division 80
CREST: Calcinosis, Raynaud’s phenomenon, esophageal dismotility, sclerodactyly, and telangiectasia
FISH: Fluorescence in situ hybridization
CRM: Centromeric retrotransposon of maize
