# Supplementary figures and images for: Total centromere size and genome size are strongly correlated in ten grass species
Source: Chromosome Res. 2012 May 3;20(4):403–12. doi: 10.1007/s10577-012-9284-1 (PMC3391362; doi:10.1007/s10577-012-9284-1)

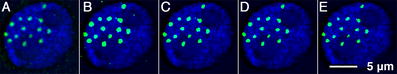

Supplement: Supplementary file 1 — Illustration of the masking protocol used to determine total centromere area. A single image from barley is shown after masking at various threshold values. CENH3 is shown in green and DAPI is shown in blue. a Original unmasked image. b–e Images shown after masking at progressively higher threshold values so that more and more of the staining is removed. The image in (d) shows the threshold chosen, which was set at one grey level above that needed to remove all non-kinetochore background staining (JPEG 8 kb) [file 10577_2012_9284_Fig6_ESM.jpg]

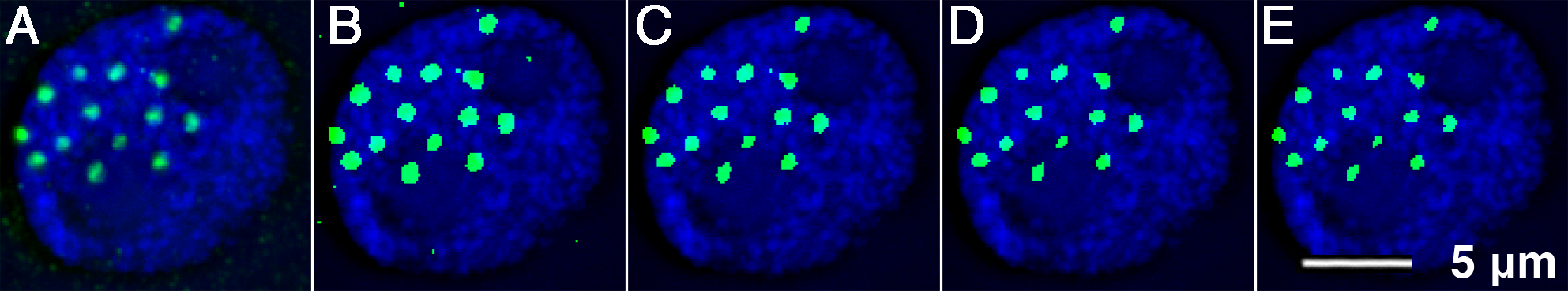

Supplement: Supplementary file 2 — High resolution image (TIFF 2,176 kb) [file 10577_2012_9284_MOESM1_ESM.tif]

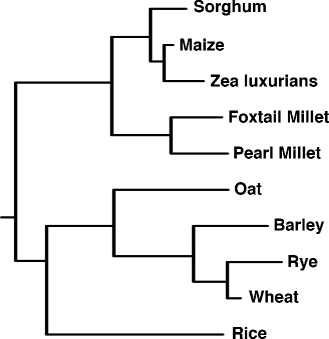

Supplement: Supplementary file 3 — Phylogenetic tree showing the relationship of the grass species chosen in this study. The phylogenetic tree was drawn using the following Newick tree: (((sorghum, 0.00950939280685307413 (maize, 0.00231520485691936832; Z. luxurians, 0.01052332533144828206), 0.00367176807056998015), 0.01041711803339982756 (foxtail millet, 0.01363691334023187443; pearl millet, 0.01520671944747864317), 0.01592780163032410726), 0.02586075002500199532 ((oat, 0.03072769607026095903 (barley, 0.01983366653899813412 (rye, 0.01449881409177665101; wheat, 0.00350123323061236674), 0.00909150693055843159), 0.02146457938246661493), 0.01807421402216232917; rice, 0.04736460143268115403), 0.00833952027397439483). The same tree was applied to perform independent contrast using the COMPARE 4.6b software (GIF 8 kb) [file 10577_2012_9284_Fig7_ESM.gif]
